# 2-Arachidonyl-lysophosphatidylethanolamine Induces Anti-Inflammatory Effects on Macrophages and in Carrageenan-Induced Paw Edema

**DOI:** 10.3390/ijms22094865

**Published:** 2021-05-04

**Authors:** Soo-Jin Park, Dong-Soon Im

**Affiliations:** 1 College of Pharmacy, Pusan National University, Busan 46241, Korea; sonicsy14@naver.com; 2 Laboratory of Pharmacology, College of Pharmacy, and Department of Biomedical and Pharmaceutical Sciences, Graduate School, Kyung Hee University, Seoul 02447, Korea

**Keywords:** lysolipid, 2-arachidonyl-lysophosphatidylethanolamine, inflammation, edema, macrophage

## Abstract

2-Arachidonyl-lysophosphatidylethanolamine, shortly 2-ARA-LPE, is a polyunsaturated lysophosphatidylethanolamine. 2-ARA-LPE has a very long chain arachidonic acid, formed by an ester bond at the *sn*-2 position. It has been reported that 2-ARA-LPE has anti-inflammatory effects in a zymosan-induced peritonitis model. However, it’s action mechanisms are poorly investigated. Recently, resolution of inflammation is considered to be an active process driven by M2 polarized macrophages. Therefore, we have investigated whether 2-ARA-LPE acts on macrophages for anti-inflammation, whether 2-ARA-LPE modulates macrophage phenotypes to reduce inflammation, and whether 2-ARA-LPE is anti-inflammatory in a carrageenan-induced paw edema model. In mouse peritoneal macrophages, 2-ARA-LPE was found to inhibit lipopolysaccharide (LPS)-induced M1 macrophage polarization, but not induce M2 polarization. 2-ARA-LPE inhibited the inductions of inducible nitric oxide synthase and cyclooxygenase-2 in mouse peritoneal macrophages at the mRNA and protein levels. Furthermore, products of the two genes, nitric oxide and prostaglandin E_2_, were also inhibited by 2-ARA-LPE. However, 1-oleoyl-LPE did not show any activity on the macrophage polarization and inflammatory responses. The anti-inflammatory activity of 2-ARA-LPE was also verified in vivo in a carrageenan-induced paw edema model. 2-ARA-LPE inhibits LPS-induced M1 polarization, which contributes to anti-inflammation and suppresses the carrageenan-induced paw edema in vivo.

## 1. Introduction

Lysophosphatidic acid is a representative lyso-type intercellular mediator that acts through G protein-coupled receptors (LPA_1-6_) [1]. Lysophosphatidylethanolamine (LPE) is another lyso-type phospholipid and has been detected in human serum at concentrations of several hundreds of ng/ml [2,3]. However, its action has not been much studied.

Previously, we have studied effects of long-chain LPEs such as 1-oleoyl LPE (1-OLE-LPE), 1-palmitoyl LPE, and 1-myristoyl LPE on intracellular Ca^2+^ ([Ca^2+^]_i_) increasing actions in PC-12 and SH-SY5Y neuronal cells, and SK-OV3 ovarian and MDA-MB-231 breast cancer cells [4,5,6,7]. These studies elucidated that LPE-induced [Ca^2+^]_i_ responses have common and dissimilar characteristics among cell types; that is, LPA_1_ involvement is common to PC-12, SH-SY5Y, and MDA-MB-231 cells, but differs in SK-OV3 cells, whereas responses to different LPE structural types differed in the cell lines.

On the other hand, analysis of plasma LPE species using a liquid chromatography-tandem mass spectrometry found 7–17 μM of total LPE in patients undergoing coronary angiography [8]. Specifically, plasma levels of polyunsaturated long chain LPEs including 22:6 LPE, 20:4 LPE, and 18:2 LPE were increased about three-fold in acute coronary syndrome subjects compared to levels in patients with normal coronary arteries [8]. Furthermore, 2-polyunsaturated acyl-LPEs, such as 2-arachidonyl LPE (2-ARA-LPE) and 2-docosahexaenoyl LPE, were found to have anti-inflammatory action in a zymosan A-induced peritonitis model [9]. Reduction of vascular leakage was found in a 2-ARA-LPE-treated group, however, its action mechanisms are not well investigated [9]. Recently, resolution of inflammation is considered to be an active process driven by M2 polarized macrophages. Therefore, we have investigated whether 2-ARA-LPE acts on macrophages for anti-inflammation, whether 2-ARA-LPE modulates macrophage phenotypes to reduce inflammation in comparison with 1-OLE-LPE, and whether 2-ARA-LPE is anti-inflammatory in a carrageenan-induced paw edema model.

## 2. Results

### 2.1. Effects of 2-ARA-LPE on Macrophage Phenotypes

**Figure 1:**
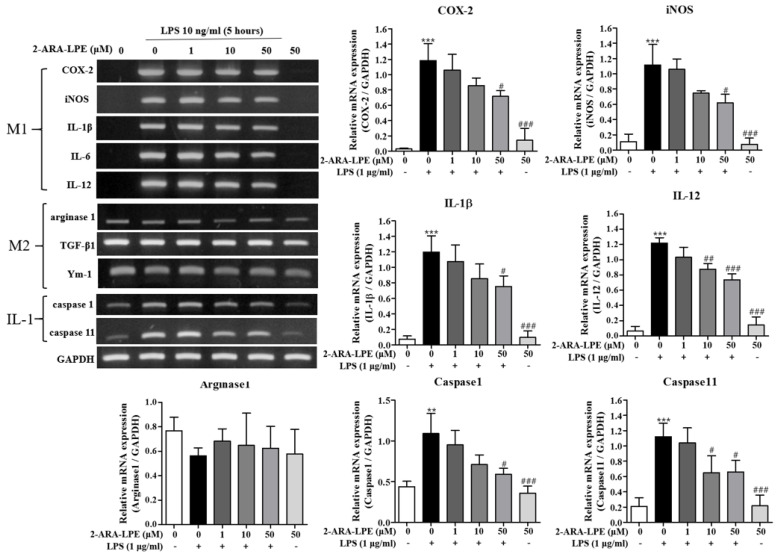
Effects of 2-ARA-LPE on the expressions of M1 and M2 marker genes in peritoneal macrophages. Mouse peritoneal macrophages were treated with the indicated concentrations of 2-ARA-LPE for 1 h, and then treated with vehicle or LPS 10 ng/mL for 5 h. RT-PCR for pro-inflammatory genes and anti-inflammatory genes was performed. The data are representative of three independent experiments. Relative mRNA levels of each gene versus glyceraldehyde 3-phosphate dehydrogenase (GAPDH) are shown as histograms. ^**^ *p* < 0.01, ^***^ *p* < 0.001 vs. the none-treated group. ^#^ *p* < 0.05, ^##^ *p* < 0.01, ^###^ *p* < 0.001 vs. the LPS-treated group.

**Figure 2:**
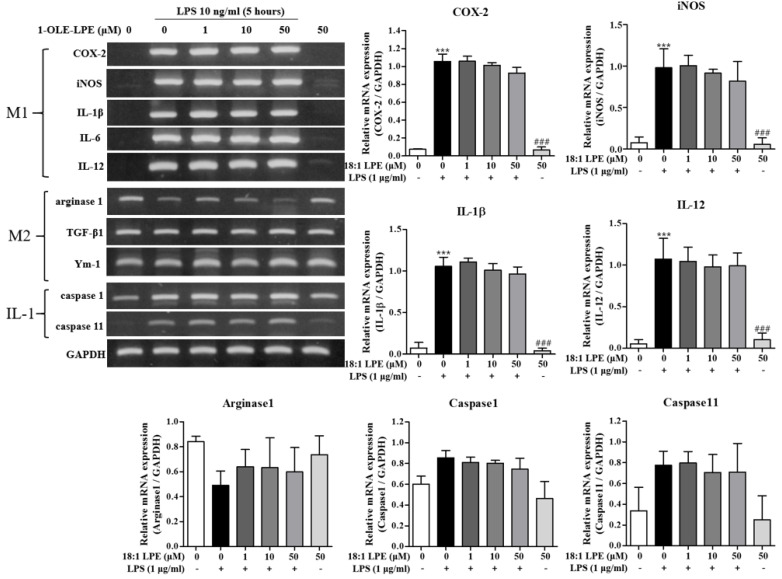
Effects of 1-OLE-LPE on the expressions of M1 and M2 marker genes in peritoneal macrophages. Mouse peritoneal macrophages were treated with the indicated concentrations of 1-OLE-LPE for 1 h, and then treated with vehicle or LPS 10 ng/mL for 5 h. RT-PCR for pro-inflammatory genes and anti-inflammatory genes was performed. The data are representative of three independent experiments. Relative mRNA levels of each gene versus GAPDH are shown as histograms. ^***^ *p* < 0.001 vs. the none-treated group. ^###^ *p* < 0.001 vs. the LPS-treated group.

We aimed to investigate if 2-ARA-LPE executes anti-inflammatory effects on lipopolysaccharide (LPS)-activated murine peritoneal macrophages. We compared effects of 2-ARA-LPE and 1-OLE-LPE on the M1 and M2 polarizations of peritoneal macrophages. As shown in Figure 1, isolated peritoneal macrophages expressed M2 marker genes, such as arginase-1, TGF-β, and Ym-1, but not M1 marker genes, such as cycolooxygenase-2 (COX-2), inducible nitric oxide (iNOS), IL-1β, IL-6, and IL-12. However, after stimulation with lipopolysaccharide (LPS; a cell wall component of Gram-negative bacteria) macrophage phenotype changed; that is, they strongly expressed M1 marker genes, such as, COX-2, iNOS, IL-1β, IL-6, and IL-12 (Figure 1). Furthermore, the representative M2 marker gene, arginase-1, was down-regulated after LPS stimulation (Figure 1) [10]. These observations show that the isolated peritoneal macrophages were M2 polarized, and that the LPS-stimulated macrophages were M1 polarized [10]. Using this macrophage polarization phenomenon, the effects of the 2-ARA-LPE and 1-OLE-LPE were assessed. 2-ARA-LPE suppressed LPS-induced inductions of iNOS, COX-2, IL-1β, IL-6, and IL-12 expression, but did not induce arginase-1 expression (Figure 1). Expression of inflammasome-related caspase-1 and 11 was also measured. LPS induced inductions of caspase-1 and 11, and 2-ARA-LPE also suppressed the inductions in a concentration dependent manner (Figure 1). However, 1-OLE-LPE did not affect the expressions of M1 and M2 marker genes, suggesting specificity of 2-ARA-LPE (Figure 2). Also, 1-OLE-LPE did not affect the expressions of caspase-1 and 11, in contrast to 2-ARA-LPE (Figure 2).

### 2.2. Effects of 2-ARA-LPE on COX-2 and iNOS Expression

**Figure 3:**
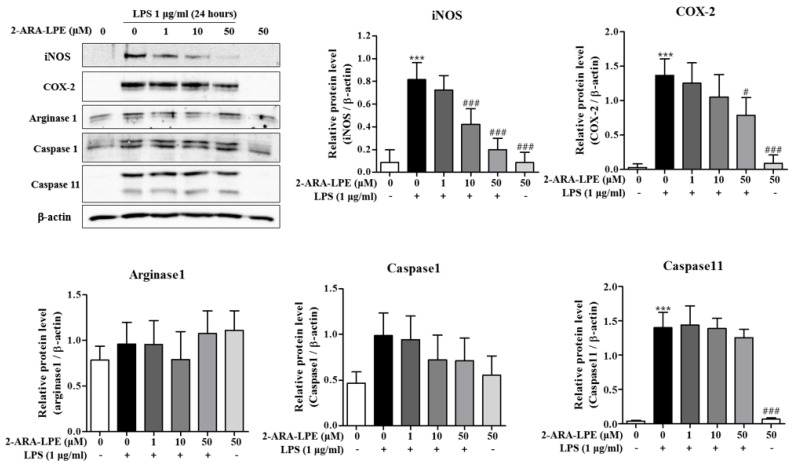
Effect of 2-ARA-LPE on the protein expressions of COX-2, iNOS, arginase-1, caspase-1, and caspase-11 in macrophages. Mouse peritoneal macrophages were treated with the indicated concentrations of 2-ARA-LPE for 1 h, and then treated with LPS 1 μg/mL for 24 h. Western blotting was conducted on cell lysates. The data are representative of three independent experiments. Relative protein levels of each protein versus β-actin are shown as histograms. ^***^ *p* < 0.001 vs. the none-treated group. ^#^ *p* < 0.05, ^###^ *p* < 0.001 vs. the LPS-treated group.

**Figure 4:**
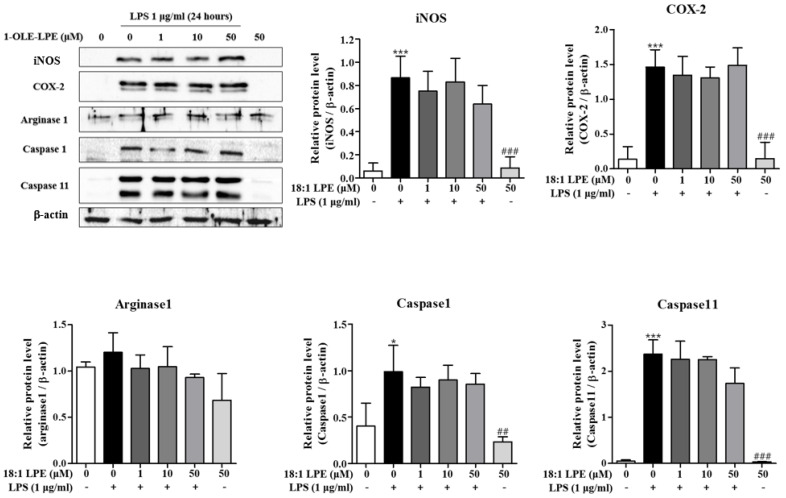
Effect of 1-OLE-LPE on the protein expressions of COX-2, iNOS, arginase-1, caspase-1, and caspase-11 in macrophages. Mouse peritoneal macrophages were treated with the indicated concentrations of 1-OLE-LPE for 1 h, and then treated with LPS 1 μg/mL for 24 h. Western blotting was conducted on cell lysates. The data are representative of three independent experiments. Relative protein levels of each protein versus β-actin are shown as histograms. ^*^ *p* < 0.05, ^***^ *p* < 0.001 vs. the none-treated group. ^##^ *p* < 0.01, ^###^ *p* < 0.001 vs. the LPS-treated group.

We also examined the inhibitory effects of 2-ARA-LPE at the protein level. As shown in Figure 3, the expressions of iNOS and COX-2 protein were obviously induced by LPS. 2-ARA-LPE strongly and concentration-dependently inhibited iNOS protein induction and COX-2 protein induction (Figure 3). At concentrations 10 and 50 μM, 2-ARA-LPE had significant inhibitory effects on iNOS protein induction. COX-2 protein expression was also significantly inhibited by 2-ARA-LPE at 50 μM concentration (Figure 3). Expressions of both proteins, however, were not affected by 1-OLE-LPE (Figure 4). 2-ARA-LPE-induced suppression of inflammasome-related caspase-1 and 11 expression at mRNA level was not confirmed at protein level (Figure 1 and Figure 3), although there was a suppressive tendency on caspase-1 (Figure 3).

**Figure 5:**
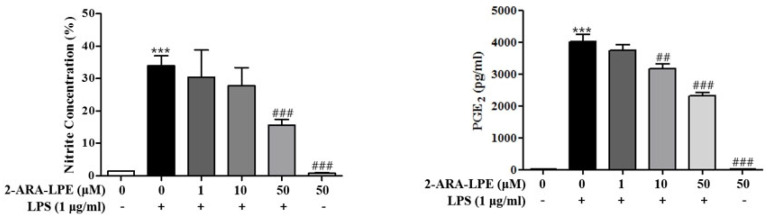
Effect of 2-ARA-LPE on production of nitrite and prostaglandin E_2_ in macrophages. Mouse peritoneal macrophages were treated with the indicated concentrations of 2-ARA-LPE for 1 h, and then treated with vehicle or LPS 1 μg/mL for 24 h. LPS-induced productions of nitrite (**A**) and PGE_2_ (**B**) were measured. Results are the means ± SDs of three independent experiments. Statistically significant at the *** *p*<0.001 levels vs. the none-treated macrophages, ^##^ *p* <0.01 and ^###^ *p* <0.001 levels vs. the LPS-treated macrophages.

Next, the inhibitory effects of 2-ARA-LPE on the expressions of iNOS and COX-2 were confirmed by measuring the products of iNOS and COX-2, that is, nitric oxide (NO) and prostaglandin E_2_ (PGE_2_), respectively. As shown in Figure 5, 2-ARA-LPE significantly and concentration-dependently inhibited LPS-induced NO production (Figure 5A), and as shown in Figure 5B, 2-ARA-LPE markedly inhibited PGE_2_ production in a concentration-dependent manner from a concentration of 10 μM, supporting the suppressive effects of 2-ARA-LPE on expressions of iNOS and COX-2.

### 2.3. Effect of 2-ARA-LPE on Carrageenan-Induced Paw Edema

**Figure 6:**
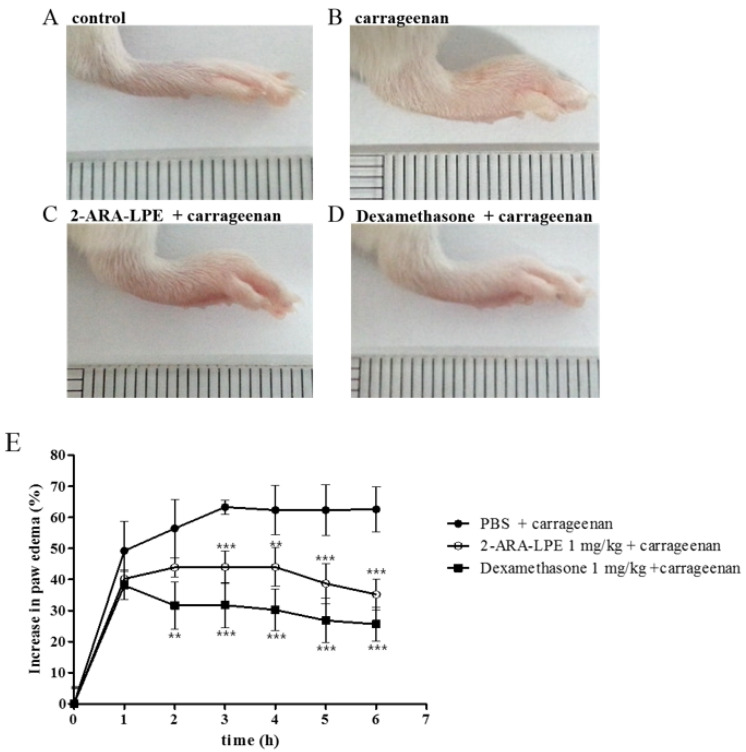
Changes in paw thickness (mm) of ICR mice. Visual representation of before (**A**) and after (**B**) injection of carrageenan in hind paw of ICR mice. Changes in paw thickness by pre-treatment with (**C**) 2-ARA-LPE (1 mg/kg) or (**D**) dexamethasone (1 mg/kg, respectively) on carrageenan-induced swelling of the mouse hind paw. (**E**) Time courses of paw edema. Data expressed as mean ± SD of n = 6 mice/group. Control: phosphate-buffered saline (PBS); Carrageenan: carrageenan-treated; 2-ARA-LPE + carrageenan: 2-ARA-LPE plus carrageenan-treated; Dexamethasone + carrageenan: dexamethasone plus carrageenan-treated. ** *p* <0.01 and *** *p* <0.001 vs. carrageenan-treated group.

In a previous study, 2-ARA-LPE was found to reduce vascular leakage in a zymosan A-induced peritonitis model [9]. In order to verify and expand the anti-inflammatory effect of 2-ARA-LPE, carrageenan-induced acute inflammatory paw edema model was employed [11]. Injection of carrageenan into the hind paw of mice resulted in edema which was assessed by paw thickness (Figure 6). After injection of carrageenan, the paw size increased 48% in one hour. Maximal effect of carrageenan was produced at three hours after injection, with 60% thickening of the paw. The swelling sustained for six hours. 2-ARA-LPE (1 mg/kg) displayed obvious suppression of the paw edema. The suppressive efficacy of 2-ARA-LPE (1 mg/kg) was similar to that of dexamethasone (1 mg/kg) (Figure 6).

**Figure 7:**
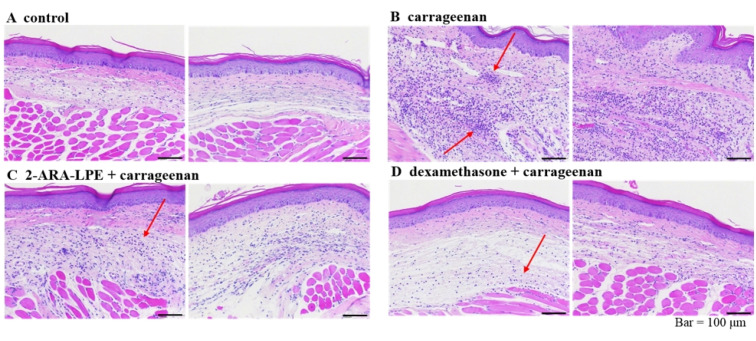
Histological examination of paw tissue sections 3 h after carrageenan injection. (**A**) Normal control, (**B**) carrageenan control without drug treatment, (**C**) treated with 2-ARA-LPE + carrageenan, (**D**) treated with dexamethasone + carrageenan. Red arrows indicate infiltrated neutrophils.

Histological analysis of tissue sections revealed reduction of cellular infiltration of immune cells in the 2-ARA-LPE-treated group. Microscopic photographs of the control stained with hematoxylin and eosin showed normal paw tissue with no signs of inflammation (Figure 7). In the carrageenan-treated group, high infiltration damage was found due to accumulation of immune cells and collection of fluid, as the arrow indicated in Figure 7B shows. However, 2-ARA-LPE (Figure 7C), and dexamethasone (Figure 7D)-treated groups showed only moderate infiltration damage. The 2-ARA-LPE-treated group showed anti-inflammatory effects similar to that of the positive control dexamethasone.

## 3. Discussion

Recent analysis of plasma lysophospholipids species using a liquid chromatography-tandem mass spectrometry found 7–17 μM of total LPE in patients undergoing coronary angiography [8]. Specifically, plasma levels of polyunsaturated very long chain LPEs including 22:6 LPE, 20:4 LPE, and 18:2 LPE were increased about three-fold in acute coronary syndrome subjects compared to levels in patients with normal coronary arteries [8]. However, the meaning of their levels has not been studied. In a previous study, the anti-inflammatory effect of 2-ARA-LPE was reported in a zymosan-induced peritonitis model [9]. In this present study, we found the inhibitory effect of 2-ARA-LPE on LPS-induced M1 polarization of macrophages in vitro as a mechanism for the anti-inflammation, although 2-ARA-LPE did not affect M2 polarization. Furthermore, we found an in vivo inhibitory effect of 2-ARA-LPE in a carrageenan-induced edema model.

One interesting finding is that 1-OLE-LPE did not induce suppressive effect on M1 polarization. We previously have studied 1-OLE-LPE as the most potent Ca^2+^-mobilizing agonist on several cell lines [4,5,6,7]. In mouse peritoneal macrophages, however, 2-ARA-LPE is anti-inflammatory but 1-OLE-LPE is not. This implies that the target of 2-ARA-LPE is different from the receptors for 1-OLE-LPE, and that there may be many different receptors for different LPE species. Further studies are necessary to elucidate the action mechanism of LPEs.

## 4. Materials and Methods

### 4.1. Materials

2-ARA-LPE was made from 1-palmitoyl 2-arachidonyl phosphatidylethanolamine by acidic hydrolysis [9]. 1-OLE-LPE was purchased from Avanti Polar Lipids (Alabaster, AL, USA). They were dissolved in absolute methanol and stored at −20 °C. All other chemicals were purchased from Sigma-Aldrich (St. Louis, MO, USA).

### 4.2. Animals

8–10 week old male C57BL/6 (19–22g) mice and 6 week old male ICR (28–31g) mice were purchased from Daehan Biolink (DBL; Seoul, Korea), housed in a laboratory animal facility at Pusan National University, and provided with food and water ad lib. The animal protocol used in this study was reviewed and approved beforehand by the Pusan National University—Institutional Animal Care Committee (PNU—IACUC) with respect to ethical and scientific care.

### 4.3. Isolation and Culture of Mouse Peritoneal Macrophages

Mouse peritoneal macrophages were isolated from the peritoneal cavity of a 3% thio-glycollate-treated C57BL/6 mouse 4 days after treatment and cultured at 37 °C in a 5% CO_2_ humidified incubator. Isolated macrophages were maintained in RPMI1640 containing 10% (v/v) heat-inactivated fetal bovine serum, 100 units/mL penicillin, 50 μg/mL streptomycin, 2 mM glutamine, and 1 mM sodium pyruvate for 18 h and then incubated in 0.5% FBS-containing media for 24 h. RNA and protein samples were prepared after 5 h or 24 h of LPS treatment (10 ng/mL or 1 μg/mL), respectively. LPEs were added 1 h before adding LPS [10].

### 4.4. Reverse Transcriptase-PCR

To determine the expressions of marker proteins of M1 or M2 polarization in macrophages by RT-PCR, first strand cDNA was synthesized with total RNA isolated using Trizol reagent (Invitrogen, USA). Synthesized cDNA products and primers for each gene were used for PCR, which was conducted using Promega Go-Taq DNA polymerase (Madison, WI, USA).

Specific primers for IL-12 (sense 5′-CAG AAG CTA ACC ATC TCC TGG TTT G-3′, antisense 5′-TCC GGA GTA ATT TGG TGC TTC ACA C-3′), TGF-β1 (sense 5′-TTG CTT CAG CTC CAC AGA GA-3′, antisense 5′-TGG TTG TAG AGG GCA AGG AC-3′), Ym-1 (sense 5′-ACT TTG ATG GCC TCA ACC TG-3′, antisense 5′-AAT GAT TCC TGC TCC TGT GG-3′), caspase1 (sense 5′-CCA GAG CAC AAG ACT TCT GAC-3′, antisense 5′-TGG TGT TGA AGA GCA GAA AGC-3′), and caspase11 (sense 5′-CTT CAC AGT GCG AAA GAA CT-3′, antisense 5′-GGT CCA CAC TGA AGA ATG TCT GGA GAA GCA TTT CA-3′) were used to amplify gene fragments. PCR was performed over 30 cycles of denaturation at 95 °C for 30 s, annealing at 55 °C for 30 s, and elongation at 72 °C for 30 s in an Eppendorf Mastcycler gradient PCR machine (Hamburg, Germany) [12]. Specific primers for arginase-1 (sense 5′-GTG AAG AAC CCA CGG TCT GT-3′, antisense 5′-CTG GTT GTC GGG GAG TGT T-3′), iNOS (sense 5′-ACC TAC CAC ACC CGA GAT GGC CAG-3′, antisense 5′-AGG ATG TCC TGA ACA TAG ACC TTG GG-3′), COX-2 (sense 5′-CCG TGG GGA ATG TAT GAG CA-3′, antisense 5′-CCA GGT CCT CGC TTA TGA TCT G-3′), and GAPDH (sense 5′-TTC ACC ACC ATG GAG AAG GC-3′, antisense 5′-GGC ATG GAC TGT GGT CAT GA-3′) were used and annealing was undertaken at 60 °C. For IL-1β (sense 5′-GGA GAA GCT GTG GCA GCT A-3′, antisense 5′-GCT GAT GTA CCA GTT GGG GA-3′) and IL-6 (sense 5′-TGG GAA ATC GTG GAA ATG AG-3′, antisense 5′-GAA GGA CTC TGG CTT TGT CT-3′), annealing was undertaken at 57 °C. Aliquots (7 µl) were electrophoresed in 1.2% agarose gels and stained with StaySafe^TM^ Nucleic Acid Gel Stain (Real Biotech Corporation, Taipei, Taiwan) [10,13].

### 4.5. Western Blot

Macrophages were harvested and resuspended in RIPA lysis buffer (GenDEPOT, Baker, TX, USA). Concentrations of proteins were determined using a BCA protein assay (ThermoScientific, Rockford, IL, USA). Proteins (30 μg) were resolved by 10% SDS-polyacrylamide gel electrophoresis and electrophoretically transferred to nitrocellulose. Membranes were blocked in Tris-buffered saline containing 0.1% Tween 20 (TBS-T) and 5% skim milk, incubated with specific primary antibodies recognizing β-actin, COX-2, iNOS, arginase-1, caspase-1, and caspase-11, and then incubated with HRP-conjugated secondary antibodies (Cell Signaling Technology, Danvers, MA, USA). Signals were developed using an enhanced chemiluminescence system (Pierce Biotechnology Inc., Rockford, IL, USA) [14].

### 4.6. Nitrites Measurement

NO production was estimated by measuring the amount of nitrite (a stable metabolite of NO) in medium using Griess reagent, as previously described [14,15]. Cells were pre-treated with different concentrations of 2-ARA-LPE for 1 h and subsequently stimulated with LPS (1 μg/ml) for 24 h. Nitrite concentrations in medium were determined using the Griess Reagent System (Promega, Madison, WI, USA).

### 4.7. PGE_2_ Production

Peritoneal macrophages were incubated with 2-ARA-LPE for 1 h and subsequently stimulated with LPS (1 μg/ml) for 24 h. Macrophage culture supernatants were harvested and immediately assayed using a PGE_2_ EIA kit (Cayman Chemical, Ann Arbor, MI, USA) [16].

### 4.8. Carrageenan-Induced Paw Edema Assay

The carrageenan-induced hind paw edema model in mice was used to assess anti-inflammatory activity [11]. ICR mice were divided into the 3 groups (*n* = 5/group). 2-ARA-LPE (1 mg/kg) or dexamethasone (1 mg/kg) dissolved in 1% DMSO in PBS was administered by intraperitoneal injection, and the solvent alone served as a vehicle control. Thirty minutes after the administration of drugs, paw edema was induced by sub-plantar injection of 50 μl of 1% freshly prepared carrageenan suspension in PBS into the left hind paw of each mouse. The right hind paw was injected with 50 μl of PBS. To gauge the extent of inflammation, paw thickness was measured before (0 h) and at intervals of 1, 2, 3, 4, and 6 h after carrageenan injection using a digital vernier caliper (Stainless Steel Digital Caliper, Find it at the Bay, Gaithersburg, MD, USA) [17].

### 4.9. Histology

After 6 h of carrageenan-induced edema, five animals from each group were euthanized. Paw samples were taken for histological examination. Sectioned tissues were stained with hematoxylin and eosin and viewed under a light microscope (Zeiss, Jena, Germany).

### 4.10. Statistical Analysis

Results are expressed as the means ± SDs of the indicated numbers of determinations. The statistical significances of differences were determined by analysis of variance (ANOVA) with turkey’s post hoc, and statistical significance was accepted for p values < 0.05. Analyses were performed using GraphPad Prism software (GraphPad Software, Inc., La Jolla, CA, USA).

## Data Availability

The study does not report any data.
